# Occasional Jerks of Local Muscle Groups as a Sign of Epilepsy

**Published:** 1910-06-04

**Authors:** 


### Neurology.

### OCCASIONAL JERKS OF LOCAL MUSCLE GROUPS AS A SIGN OF EPILEPSY.

Describing them under the heading of regional and myoclonic convulsions Muskens draws attention to the fact that if a careful inquiry is made in the routine examination of genuine epileptics it will be found that localised jerks or jerk-like phenomena often, if not frequently, occur in the previous history. As a rule these regional and myoclonic movements are not associated with loss of consciousness, but Muskens regards that as a minor form of the motor discharge, and as an important indication that a patient is an epileptic; he looks upon these regional convulsions or myoclonic jerks as a very common symptom of the pre-epileptic stage, or rather, if persistently present alone, as a special clinical variety, which, however, usually lapses into and becomes complicated with complete epileptic convulsions. After the appearance of the latter the clinical picture soon becomes indistinguishable from that of the ordinary complete case. The reason why the great frequency of this clinical variety has been overlooked is that most of the cases do not come under medical care until years after the first serious fits have begun to complicate the case, and, unless specially asked for, one will probably never hear of the jerk-like symptoms. Yet occasionally it may transpire from information given by the relatives, not by the patient himself, that years before the first fit took place these signs of latent epilepsy were already present. The patient, when dressing, would have jerks nearly every morning, or at night, while falling asleep or during the sleep; and in such vehemency that they might cause him, for instance, to drop his comb, or fall to the floor. It is a peculiarity of the lay mind, however, not to reveal this pathological condition until after the major fits have set in. Another circumstance that equally tends to hide the regional convulsions from the relatives is that, in many cases, after the appearance of major fits the local or regional jerks or convulsions disappear.

One will not rarely come across non-epileptic members in affected families who suffer from similar jerks, either during sleep or when getting up in the morning, but who have never had any complete epileptic attacks.

As to the frequency of these regional convulsions, in Musken's cases they were found present as follows:—

Regional Convulsions Present in Some Period of the Disease.

| | Female Cases | Male Cases |
|---|---|---|
| First 50 cases ... ... ... | 35 | 22 |
| Second " ... ... ... | 36 | 34 |
| Third " ... ... ... | 32 | 26 |
| | — | — |
| | 103 | 82 |

Of the 150 female cases, 38 had during a longer period, ranging from several months to years, the symptom alone present; of the 150 male cases only 21. In 43 of the 150 female cases the local jerky attacks developed shortly after the first major fit had made its appearance, and in 45 of the 150 male cases. In 22 female and 16 male patients no information on this point could be gathered. In their frequency and their typical relation to the appearance afterwards of major fits, which they gradually lead up to, it is maintained that those cases that have suffered from regional convulsions preceding the major fits should be considered as a distinct group. Muskens has been compelled to consider them as a separate clinical subdivision of epilepsy, because this class of cases singles itself out immediately an endeavour is made to classify the clinical forms of epilepsy after their manner of development.

It is not necessary here to describe the different forms of jerky movements of limbs and trunk resultant upon regional muscular contractions, but it may be said that contraction of the trunk muscles (either flexion or extension), or a general adduction or abduction of all the joints, particularly of one or both of the upper extremities, are the commonest forms. Gowers describes them as bilateral, multimuscular contractions. As such they may resemble choreiform contractions, although the latter are always distinguished by their quasi-voluntary character, and are hardly ever of such a sudden nature. Rarely are they localised in one single muscle or part of a muscle.

In general one may say that:—

1. The localisation of the regional convulsions is such that no voluntary muscle is excluded. The upper extremities are affected the most. Besides cases with asymmetrical distribution there are others of symmetrical occurrence.

2. There are certain periods of the daily routine during which the regional convulsions are prone to appear—namely, when getting up in the morning; the moment of going to sleep.

3. Besides cases where intentional movements reinforce the regional convulsions cases also occur where this is not so. There are cases where the regionary convulsions manifest themselves exclusively during sleep, and, again, cases where the convulsions are less prone to appear during the sleep of the patient. It is doubtful whether subsidence or disappearance during sleep could be supposed to have any influence on the diagnosis.

4. In practically every one of the cases of purely regional convulsions actual epilepsy had occurred in the family.

5. Regionary convulsions in some cases will disappear, while in many others they will be accentuated, during pregnancy.

6. In slight cases of distinct regional convulsion, as also in those attended with major epileptic fits, a prophylactic treatment with bromides is often found successful. Purely regional convulsions, if strongly developed, are far more obstinate to treatment; in some instances the zinc treatment was productive of better results.

7. A horizontal posture reinforces the regional convulsions.

8. Constipation very clearly aggravates the regional convulsions.

9. Physical deterioration does *not* complicate purely regional convulsions. From the observation of different cases it is clearly manifest that mental disturbance does not set in until shortly after the first complete fit occurs, years of the most vehement regional convulsions proving powerless in that respect. On the other hand, a compensatory relation can frequently be discovered between the regional convulsions and the complete epileptic fits.

This compensation may be twofold. As soon as a distinct case of regional convulsions has developed into the periodic appearance of major epileptic attacks, these convulsions from first appearing every morning, will slip into being a premonitory symptom of the major fit, eventually to disappear entirely, so that it is quite possible that the character of the case, from first being one of constantly recurring or even continuous regional convulsions, will often merge into one characterised by complete periodic attacks, free of regional convulsions, but subject to mental disturbances. Muskens strongly believes that the appearance of major fits should here be regarded less as the commencement of the disease than as a terminal result.

